# Comparison of Muscle Activation between Variable Resistance Training and Free Weight Training: A Systematic Review and Meta-Analysis

**DOI:** 10.5114/jhk/202810

**Published:** 2025-09-23

**Authors:** Jie Li, Zongwei Chen, Mingjun Gong

**Affiliations:** 1 School of Sports Training, Tianjin University of Sport, Tianjin, China.; 2 School of Physical Education and Sports Science, South China Normal University, Guangzhou, Guangdong, China.; 3 Department of Physical Education and Sport, Faculty of Sport Sciences, University of Granada, Granada, Spain.

**Keywords:** contraction of muscle, electromyography, strength training

## Abstract

Variable resistance training (VRT) can address biomechanical limitations of free weight training (FWT) and enhance performance, but its effect on muscle activation remains unclear. This study aimed to compare muscle activation between VRT and FWT, focusing on the impact of variable resistance load proportions and contraction phases in VRT. A systematic review was conducted using PubMed, Scopus, SPORTDiscus, and Web of Science, including studies of healthy adults with at least six months of training experience performing multi-joint exercises. Muscle activation was measured using surface electromyography (sEMG), and Standardized Mean Differences (SMD) were calculated using a random-effects model. The meta-analysis of 11 studies found no significant overall difference between VRT and FWT (SMD = 0.07; p = 0.493). However, VRT showed greater activation during the concentric phase than both the eccentric phase (SMD = 1.40; p < 0.001) and the FWT’s concentric phase (SMD = 0.32; p = 0.033). Higher variable resistance load proportions in VRT also resulted in greater activation than FWT at equivalent loads (SMD = 0.38; p = 0.036). Thus, under the same load, VRT significantly improves concentric-phase muscle activation over FWT, with higher variable resistance loads providing even greater benefits.

## Introduction

Free weight training (FWT) has long been a cornerstone of resistance training programs and is widely used by athletes to enhance muscle strength and performance (Cui et al., 2024; Schoenfeld, 2010). However, FWT presents several limitations (Kompf and Arandjelović, 2017). One of the key limitations is that the resistance in FWT remains constant throughout the entire movement, which means it cannot be adjusted in real-time during the movement. Due to biomechanical constraints, the body's levers and joint angles change at different stages of the movement. This leads to the muscles having a greater mechanical advantage in certain ranges of motion while experiencing a reduced load in other ranges (Tsoukos et al., 2024). Variable resistance training (VRT), as an extension of traditional FWT, offers dynamically changing resistance throughout the movement, allowing the training load to effectively target the athlete at every phase of the exercise (Kompf and Arandjelović, 2016). Furthermore, it enhances the athlete's ability to generate force and power at the end of the movement, which may increase the adaptability of the neuromuscular system (Shi et al., 2024). VRT has gained significant attention for its proven effectiveness in improving athletes' overall performance (Andersen et al., 2022). Typically building on FWT, VRT incorporates elastic bands or chains as additional resistance, allowing athletes to experience varying loads throughout the entire range of motion during a given exercise (e.g., bench press or squat), thereby addressing the limitations inherent in traditional resistance training (Walker et al., 2013).

Existing studies and meta-analyses have extensively explored the superiority of VRT in improving strength and power among athletes compared to FWT (Shi et al., 2022). Furthermore, muscle activation, as a key indicator for assessing the degree of muscle involvement during resistancetraining (Gandevia, 2001), allows for the comparison of the effects of different training methods on muscle stimulation. This, in turn, helps to more efficiently achieve goals such as strength gains, muscle hypertrophy, and athletic performance enhancement (Moritani and deVries, 1979). However, there is a lack of relevant meta-analyses investigating whether VRT can induce greater muscle activation than FWT. Muscle activation levels during the movement are typically measured through surface electromyography (sEMG) (Valentin and Zsoldos, 2016) which records and analyzes the electrical activity of skeletal muscles and nerves to evaluate the functional state of the neuromuscular system. The electromyography (EMG) readings obtained via sEMG provide a detailed view of muscle activation patterns during the movement (Phinyomark et al., 2014), making it a critical tool for investigating skeletal muscle activation during voluntary movements. This information is crucial for developing effective training programs and enhancing athletic performance (Stronska et al., 2022). Additionally, higher levels of muscle activation can achieve better training outcomes within the same training duration (Stastny et al., 2017).

Previous meta-analyses have compared muscle activation levels between VRT and isokinetic training, with results indicating no significant differences between them (Aboodarda et al., 2016). However, isokinetic training is often limited to single-joint exercises. In contrast, multi-joint exercises are more effective at activating multiple muscle groups, which enhances the overall performance of athletes (Schoenfeld, 2010). Conversely, VRT is easier to implement and has broader applicability, making it more valuable for practical training purposes (Andersen et al., 2022). Therefore, comparing muscle activation levels between VRT and FWT can provide valuable insights for practical training applications.

Nijem (2016) found that in trained males, FWT was more effective in promoting gluteus maximus activation compared to VRT (combined with chains and free weights) in the deadlift. Ebben and Jensen (2002) found that in national collegiate athletic association division I athletes, a squat exercise using chains as VRT did not lead to greater quadriceps muscle activation compared to FWT. The discrepancies in results between studies may be attributed to differences in participants’ characteristics (Moritani and deVries, 1979). Some studies included trained individuals (Andersen et al., 2015; Nijem et al., 2016), while others involved untrained participants (Aboodarda et al., 2011a; Peltonen et al., 2014), and many focused on rehabilitation after injury rather than on healthy participants. Additionally, most studies have been limited to single-joint exercises (Aboodarda et al., 2011b), lacking detailed analysis of multi-joint training. It is evident that there is still ongoing debate regarding the comparison of muscle activation between VRT and FWT, and relevant meta-analyses on this topic are lacking.

The goal of this study was to explore the differences in muscle activation levels between VRT and FWT, with a focus on further refining the research by considering participants’ characteristics and training types. This research centers on the muscle activation levels of the primary muscles involved during exercise, designating individuals carrying out VRT as the experimental group, while those following FWT as the control group. By comparing the muscle activation differences between the two groups during the same movement, this study aimed to provide theoretical support for the application of VRT. Additionally, the current study conducted subgroup analyses to examine the impact of different muscle contraction phases and varying levels of the variable resistance load in VRT on muscle activation. Based on previous studies (Andersen et al., 2015; Shi et al., 2024; van den Tillaar et al., 2022), this study hypothesized that without specifying the variable resistance load, VRT and FWT would show no significant difference in muscle activation. However, under the same load, VRT would demonstrate higher muscle activation levels than FWT when the proportion of the variable resistance load was higher. Additionally, VRT would exhibit significantly greater muscle activation during the concentric phase.

## Methods

This study was conducted in strict accordance with the Preferred Reporting Items for Systematic Reviews and Meta-Analyses (PRISMA) guidelines. Additionally, the study was registered in the PROSPERO database under the registration number CRD42024550030 (date of registration: 21 September 2024). Each step of the research process was carried out independently by two authors (J.L. and Z.C.), who then cross-checked their work. In the event of discrepancies, the issue was referred to a third author (M.G.) for resolution. The raw data and related supplementary material of this manuscript can be accessed through the link: https://doi.org/10.5281/zenodo.13370681.

### Search Strategy

Literature search for this study commenced on August 20, 2023, with monthly updates. The databases searched included PubMed, Web of Science, Scopus, and SPORTDiscus. To ensure thorough and precise results, wildcard operators (AND/OR) and relevant keywords were used. PubMed’s search query served as the base template, which was adapted to each database using the Polyglot Search Translator Tool (Kung, 2022). The PubMed query was: ((“Elastic band”) OR (“Elastic”) OR (“Rubber band”) OR (“Chain”)) AND ((“Traditional resistance training”) OR (“Variable resistance”) OR (“Accommodating resistance”) OR (“Elastic resistance”) OR (“Chain resistance training”)) AND ((“EMG”) OR (Electromyography) OR (Muscle electrical signal) OR (Muscle activation)). Retrieved articles were organized using Endnote 21.4. Conference papers, systematic reviews, non-English publications, email-based articles, and those with only abstracts and no full-text availability were excluded.

### Inclusion and Exclusion Criteria

The detailed inclusion and exclusion criteria for the articles are presented in supplementary material. The inclusion criteria for participants in the selected studies were as follows: (I) participants should be between 18 and 65 years old, (II) participants must be healthy with no history of disease or injury; (III) participants must have at least six months of training experience. The criteria for the training methods included: (I) VRT must be based on FWT; (II) VRT must use elastic bands or chains as resistance or assistance; (III) blood flow restriction bands must not be used during the training process; (IV) a familiarization session must be conducted before formal training; (V) training must be performed on a stable surface and in a non-special environment; (VI) exercises must be completed within the full range of motion. For the control group: the study must compare muscle activation differences between VRT (experimental group) and FWT (control group). Regarding outcome measures: (I) the study must record EMG signals or muscle activation levels using sEMG; (II) the muscles recorded must include the agonist muscles during the training process; (III) measurements must be taken during the exercise. If any data were missing from the articles, their authors were contacted via email for clarification. Articles that still lacked the necessary information were excluded from the study.

### Data Extraction

Data from the included studies were extracted using Microsoft Excel (version 2019), capturing details such as the authors, the publication year, participants’ characteristics (number, age, gender, body height, body mass, and training level), training methods (exercise type, load intensity, tools used in VRT, and proportions of variable resistance and free weight loads), and test variables (target muscles, sEMG standardization methods, equipment used, and key findings). Muscle activation data measured by EMG in both VRT and FWT were recorded as outcome indicators, and the mean values and standard deviations (SD) were extracted for subsequent analysis. If multiple articles originated from the same raw data, data were extracted only from the most comprehensive report. For graphical data not explicitly provided in tables, WebPlotDigitizer (Drevon et al., 2017) was used to extract data, which were then imported into Stata 16.0 for further analyses.

### Quality Assessment and Risk of Bias Evaluation

The quality of the included studies was assessed using the PEDro scale (Cashin and McAuley, 2020). The original PEDro scale consists of 11 scoring criteria; however, items 5–7, which require double-blinding, were deemed difficult to apply to exercise training trials. After consulting previous research (Baz-Valle et al., 2021), two authors (J.L. and Z.C.) agreed to exclude these items and adapted the PEDro scale accordingly. Therefore, this study employed a modified version of the PEDro scale, which included 8 criteria (with the first item not scored). Each criterion was scored as 1 point if met and 0 points if not, resulting in a maximum score of 7 points. Based on previous literature (Kassiano et al., 2022), the included studies were categorized as follows: 6–7 points (excellent quality), 5 points (good quality), 4 points (moderate quality), and 0–3 points (low quality). Studies with low-quality ratings were excluded from the analysis.

Due to the intricate nature of muscle activation and EMG signal analysis, along with the inherent difficulties in evaluating these methodologies, we utilized the work of Richardson et al. (2020) who developed a specialized EMG research quality assessment tool. This tool comprised 13 methodological criteria that critically examined various aspects of the EMG measurement process. Each criterion fulfilled was awarded 1 point, with the overall score indicating the risk of bias: a score above 80% (> 10 points) indicated low risk, 70–80% (10 points) indicated moderate risk, and below 70% (0–9 points) indicated high risk.

### Statistical Analyses

Given the variability in the muscle electrical signal research methods used across the included studies, this study employed standardized mean differences (SMD) as the effect size measure. Based on previous research (Hopkins et al., 2009), SMD results were categorized as follows: SMD < 0.2 (trivial), 0.2 ≤ SMD < 0.5 (small), 0.5 ≤ SMD < 0.8 (modest), and SMD ≥ 0.8 (large). Considering the multiple studies involved and the potential internal and external heterogeneity, an inverse-variance random-effects model was used for the meta-analyses. Heterogeneity was assessed using the I^2^ statistic (Higgins and Thompson, 2002), with the following criteria: I^2^ < 25% (low heterogeneity), 25% ≤ I^2^ ≤ 75% (moderate heterogeneity), and I^2^ > 75% (high heterogeneity). A 95% confidence interval (CI) was employed, with significance determined if the CI did not include zero. The number of participants (n), publication year (years), and outcome measures (means ± SD) from the included studies were recorded in Microsoft Excel (version 2019) and analyzed using Stata 16.0 software. For the analysis of results of the included studies, publication bias was assessed using the Egger test, and sensitivity analyses were conducted for the overall comparison of muscle activation between VRT and FWT, as well as for subgroups with moderate or high heterogeneity in the subgroup analysis. These sensitivity analyses were performed to evaluate the potential impact of publication bias and high heterogeneity on the results, thereby ensuring the stability of the study findings (Greenland, 1987).

## Results

### Literature Search

**Figure 1:**
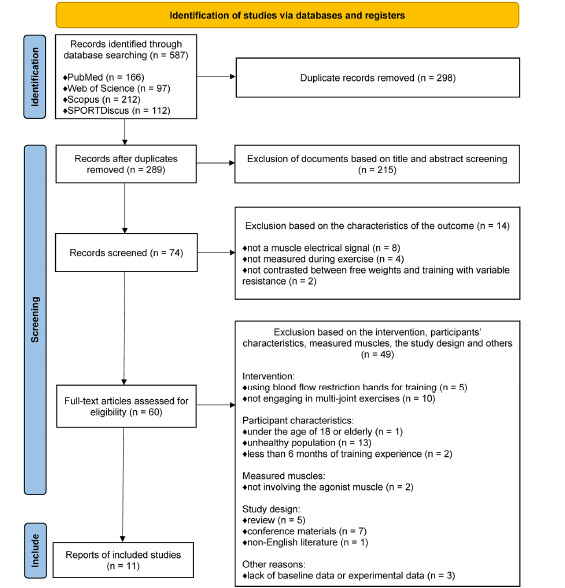
PRISMA flow diagram.

In this systematic review and meta-analysis, a total of 587 studies were initially identified. After excluding 298 duplicate records, a preliminary screening based on titles and abstracts resulted in the exclusion of 215 additional studies. Further 14 studies were excluded after reviewing the outcome measurement characteristics. These studies were not included due to reasons such as not measuring muscle electrical signals, measuring results outside the training period, or failing to compare VRT with FWT. The remaining 60 studies were subjected to full-text review, and 49 were excluded based on criteria such as participants’ characteristics, interventions, and the study design. Specifically, one study was excluded due to participants being younger than 18 or categorized as elderly, 13 studies involved unhealthy populations, and two studies were removed as participants had less than six months of training experience. Regarding intervention criteria, five studies were excluded for using blood flow restriction bands, and 10 were removed for not involving multi-joint exercises. In terms of the study design, five review articles, seven conference papers, and one non-English publication were excluded as they did not meet the inclusion criteria. Additionally, three studies were excluded due to a lack of baseline or experimental data. After the rigorous selection process, only 11 studies met the eligibility criteria and were included in the meta-analysis. Detailed search and selection information can be found in Figure 1.

### Participants’ Characteristics

Among the 11 studies included, a total of 177 participants were analyzed, comprising 100 males and 77 females. One study (Ebben and Jensen, 2002) included 5 male and 6 female participants. All participants were healthy adults with no history of exercise-related injuries or other health conditions, and all had more than six months of training experience. Additionally, one study (van den Tillaar et al., 2022) did not specify the age characteristics of 12 male participants. Upon contacting the authors, it was confirmed that all participants were over 18 years old and were not elderly. Detailed participants’ characteristics are provided in Table 1.

**Table 1:** Participants’ basic characteristics.

Study (authors, years)	Number of subjects	Gender (M/F)	Age (years)	Body mass (kg)	Body height (cm)	Training experience
(Nijem et al., 2016)	13	M	24.0 ± 2.1	87.0 ± 10.6	179.3 ± 4.8	≥ 6 months
(Andersen et al., 2015)	32	F	24.0 ± 5.0	67.0 ± 8.5	167.0 ± 6.5	≥ 6 months
(Saeterbakken et al., 2016)	20	F	23.3 ± 2.6	65.3 ± 8.5	168.0 ± 6.0	4.6 ± 2.1 years
(Andersen et al., 2020)	16	M	22.8 ± 1.7	84.0 ± 8.7	183.8 ± 6.7	2.5 ± 1.5 years
(Andersen et al., 2016)	19	F	22.3 ± 1.8	65.3 ± 9.2	162.0 ± 22.6	4.1 ± 2.2 years
(Heelas et al., 2021)	15	M	28.7 ± 9.3	92.5 ± 15.1	180.0 ± 90.0	≥ 1 year
(Andersen et al., 2019)	15	M	23.3 ± 2.2	82.8 ± 11.1	182.0 ± 6.0	3.9 ± 1.9 years
(Ebben and Jensen, 2002)	11	6 F, 5 M	19.1 ± 1.7	74.9 ± 5.6	178.0 ± 4.3	≥ 1 year
(Israetel et al., 2010)	10	M	19.8 ± 1.4	92.3 ± 20.4	177.5 ± 8.8	≥ 2 years
(van den Tillaar et al., 2022)	12	M	between 18 and 65	84.3 ± 13.5	179.0 ± 5.0	≥ 1 year
(Shi et al., 2024)	14	M	20.5 ± 0.9	82.8 ± 12.9	188.5 ± 8.5	≥ 1 year

Data are presented as means ± standard deviation. F indicates female; M, male FW indicates free weight; FW + Chain, variable resistance training method for free weights connected with the chain; FW + EB, variable resistance training method based on free weight training with an elastic band; RM, repetition maximum; Standardization, the load of variable resistance training was consistent with that of free weight training; VRT (more), variable resistance training uses a higher training load than free weight training

### Training Characteristics

All 11 studies included in the analysis (*n* = 177) required participants to refrain from resistance training for 24 h before formal testing. In terms of exercise types, six studies (*n* = 106) utilized squats, four studies (*n* = 59) involved deadlifts, and one study (*n* = 12) focused on bench presses (van den Tillaar et al., 2022). Regarding VRT methods, three studies (*n* = 36) (Ebben and Jensen, 2002; Nijem et al., 2016; van den Tillaar et al., 2022) employed chains for training, while nine studies (*n* = 152) used elastic bands. One study (*n* = 11) (Ebben and Jensen, 2002) utilized both elastic bands and chains as separate VRT methods, and one study (*n* = 15) (Andersen et al., 2019) used elastic bands attached above a barbell as assistance during training. All studies took place on stable surfaces and in non-specialized environments. Table 2 provides a detailed breakdown of training characteristics of the included studies.

**Table 2:** Training characteristics.

Study (authors, years)	Exercise	Training intensity	Training comparison VRT(Percentage of free weight load and variable resistance load) vs. FWT	Standardize and measuring load
(Nijem et al., 2016)	Deadlift	1RM (85%)	FW (80%) + Chain (20%) vs. FW	Standardization Force plate
(Andersen et al., 2015)	Squat	6RM	FW (69%) + EB (31%) vs. FW	Standardization Force plate
(Saeterbakken et al., 2016)	Squat	6RM	FW (73%) + EB (27%) vs. FW	Standardization Force plate
(Andersen et al., 2020)	Deadlift	2RM	FW (75%) + EB (25%) vs. FW	VRT (more) Force plate
(Andersen et al., 2016)	Squat	6RM	FW (61%/27%) + EB (39%/73%) vs. FW	Standardization Force cell
(Heelas et al., 2021)	Deadlift	1RM (50%)	FW (80%/70%) + EB (20%/30%) vs. FW	Standardization Force cell
(Andersen et al., 2019)	Deadlift	2RM	FW (79%/59%) + EB (21%/41%) vs. FW	Standardization Force cell
(Ebben and Jensen, 2002)	Squat	8RM	FW (90%) + Chain (10%) vs. FW FW (90%) + EB (10%) vs. FW	Standardization Force plate
(Israetel et al., 2010)	Squat	100kg	FW (75%) + EB (25%) vs. FW	Standardization Force plate
(van den Tillaar et al., 2022)	Bench press	1RM (85%)	FW (76%) + Chain (24%) vs. FW	Standardization Force plate
(Shi et al., 2024)	Squat	3RM	FW (80%/60%) + EB (20%/40%) vs. FW	Standardization Force plate

### EMG Measurement Characteristics

All the included studies collected EMG data using sEMG, with EMG calculation methods encompassing the root mean square (RMS), mean EMG, and integrated EMG. Two studies (*n* = 63) evaluated muscle electrical signals under maximum voluntary isometric contraction (MVIC) conditions. In this meta-analysis, the EMG data were processed and analyzed to evaluate muscle activation levels. Among the studies, three studies (*n* = 81) compared muscle activation differences between VRT and FWT, while seven studies (*n* = 96) expanded upon these by comparing muscle activation across different contraction phases. Four studies (*n* = 63) compared muscle activation at varying VRT load proportions, and three studies (*n* = 48) analyzed both contraction phases and VRT load proportion differences. Detailed EMG measurement characteristics, along with the names of the muscles measured, are presented in Table 3.

**Table 3:** Muscle activation test characteristics.

Study (authors, years)	EMG calculation method	Measuring muscle	Outcome (Differences in Muscle activation)
(Nijem et al., 2016)	Average EMG	GM, ES, and VL	FW + Chain < FW
(Andersen et al., 2015)	Integrated EMG (MVIC)	VL, VM, and RF	No significant differences
(Saeterbakken et al., 2016)	RMS	VL, VM, RF, and BF	No significant differences
(Andersen et al., 2020)	RMS (MVIC)	GM, S, BF, ES, VL, and VM	No significant differences
(Andersen et al., 2016)	RMS	VL, VM, RF, and BF	High proportion variable resistance loads of elastic band resistance is better, concentric phase of muscle contraction: FW + EB > FW
(Heelas et al., 2021)	RMS (MVIC)	GM, VL, VM, S, and MG	No significant differences
(Andersen et al., 2019)	RMS	ES, GM, BF, S, and VL	High proportion of elastic band resistance is better
(Ebben and Jensen, 2002)	Integrated EMG	VL, VM, BF, and S	No significant differences
(Israetel et al., 2010)	Integrated EMG	VL	Concentric phase of muscle contraction: FW + EB > FW
(van den Tillaar et al., 2022)	RMS	PJ, LD, and AD	Concentric phase of muscle contraction: FW + EB > FW
(Shi et al., 2024)	RMS	VL, VM, and RF	Concentric phase of muscle contraction: FW + EB > FW, high proportion of elastic band resistance is better

AD indicates anterior deltoid; BF, biceps femoris; EMG, electromyography; ES, erector spinae; FW, free weight; GM, gluteus maximus muscle; LD, lateral deltoid; MVIC, maximal voluntary isometric contraction; MG, medial gastrocnemius; PJ, pectoralis major muscle; RF, rectus femoris; RMS, root mean square; S, semitendinosus; VL, vastus lateralis; VM, vastus medialis

### Quality and Risk of Bias Assessment

Quality assessment of the included studies was conducted using the PEDro scale, with results summarized in supplementary material. Of the studies evaluated, one study scored 6 (excellent quality), six studies scored 5 (good quality), and the remaining four scored 4 (moderate quality), with a score range of 4–6 and an average score of 4.72 ± 0.64.

The EMG testing methods of each study were assessed for bias using an EMG method quality assessment table, with detailed results shown in supplementary material. The evaluated studies scored between 10 and 11, accounting for 76.92–84.61% of the total score. Six studies scored 11 (low risk), and five scored 10 (moderate risk), with an average score of 10.54 ± 0.52.

### Comparison of Muscle Activation between VRT and FWT

**Figure 2:**
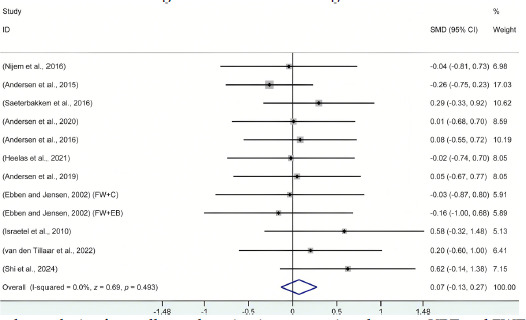
Forest plot analysis of overall muscle activation comparison between VRT and FWT. All data were calculated based on a random effects model. CI indicates confidence interval; FW+C, free weights combined with chains were used for variable resistance training; FW+EB; free weights combined with elastic bands were used for variable resistance training; SMD, standardized mean difference

After organizing the data from the 11 included studies, the forest plot analysis, as shown in Figure 2, compared muscle activation outcomes between VRT and FWT participants. Across the full range of motion, without considering the variable resistance load proportions, there was no significant difference between VRT and FWT (I^2^ = 0.0%; *df* = 11; SMD = 0.07; 95% CI: −0.13 to 0.28; *z =* 0.69; *p* = 0.493). According to previous literature (Egger et al., 1997), publication bias assessment is generally applicable in systematic reviews with more than 10 studies. Since the number of studies in the subgroups did not meet this requirement, publication bias was only evaluated for the overall analysis. The results of the Egger test did not indicate any significant publication bias (*t =* 1.79; *p* = 0.103). Additionally, sensitivity analysis revealed that after the sequential removal of individual studies, all results remained within the original 95% CI, indicating a high level of stability for the above conclusions. Detailed sensitivity analysis data, along with the Egger test data, can be found in the supplementary material.

### Different Proportions of Variable Resistance Load in VRT

**Figure 3:**
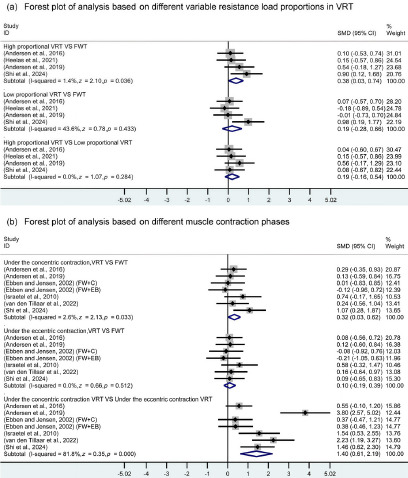
Forest plots for subgroup analysis. All data were calculated based on a random effects model. CI indicates confidence interval; FWT, free weight training; FW+C, free weights combined with chains were used for variable resistance training; FW+EB; free weights combined with elastic bands were used for variable resistance training; High proportional VRT, variable resistance training was carried out under high proportion variable resistance load; Low proportional VRT, variable resistance training was carried out under low proportion variable resistance load; SMD, standardized mean difference; VRT, variable resistance training

Among the included studies, three articles that involved VRT variable resistance loads were categorized for analysis based on the proportion of the variable resistance load within the overall training resistance. These were classified into VRT with a high variable resistance load proportion and VRT with a low variable resistance load proportion, as shown in Table 2, with the subgroup analysis forest plot in Figure 3. Data analysis revealed that muscle activation was higher in high proportion variable resistance load VRT compared to FWT with the same training load (I^2^ = 1.4%; *df =* 3; SMD = 0.38; 95% CI: 0.03 to 0.74; *z* = 2.10; *p* = 0.036). In contrast, no significant difference in muscle activation was observed between low proportion variable resistance load VRT and FWT (I^2^ = 43.6%; *df =* 3; SMD = 0.19; 95% CI: −0.28 to 0.66; *z* = 0.78; *p* = 0.433). Additionally, there was no significant difference in muscle activation between high proportion variable resistance load and low proportion variable resistance load VRT (I^2^ = 0.0%; *df =* 3; SMD = 0.19; 95% CI: −0.16 to 0.54; *z =* 1.07; *p* = 0.284).

### Different Muscle Contraction Phases

Data from seven studies were categorized based on different muscle contraction phases during exercise, dividing them into concentric and eccentric phases, with subgroup forest plot analysis shown in Figure 3. Analysis indicated that during the concentric phase, muscle activation in VRT was higher than in FWT (I^2^ = 2.6%; *df* = 6; SMD = 0.32; 95% CI: 0.03 to 0.62; *z* = 2.13; *p* = 0.033). In the eccentric phase, no significant difference was found between VRT and FWT (I^2^ = 0.0%; *df* = 6; SMD = 0.10; 95% CI: −0.19 to 0.39; *z* = 0.66; *p* = 0.512). Within VRT, muscle activation during the concentric phase was higher than during the eccentric phase (I^2^ = 81.8%; *df* = 6; SMD = 1.40; 95% CI: 0.61 to 2.19; z = 3.50; *p* < 0.001), though results showed high heterogeneity. However, sensitivity analysis revealed that after sequentially removing individual studies, all results remained within the original 95% confidence interval, indicating a high level of robustness for this conclusion. Detailed sensitivity analysis data can be found in the supplementary material.

## Discussion

This meta-analysis aimed to compare the differences in muscle activation between VRT and FWT, with a subgroup analysis conducted based on the muscle contraction phases during training and the varying proportions of resistance in VRT. The results indicated no significant difference in muscle activation levels between VRT and FWT during the entire movement when the variable resistance load proportion was not specified. However, VRT showed superior muscle activation during the concentric phase compared to FWT. Additionally, increasing the proportion of the variable resistance load (e.g., elastic bands or chains) in VRT led to enhanced muscle activation. The conclusions of this study were consistent with the hypotheses. However, this study focused only on the muscles with the highest activation levels during the squat, deadlift, and bench press exercises. Further research is needed to determine the optimal proportion of variable resistance in VRT to enhance muscle activation and explore whether VRT can outperform FWT under other conditions.

### Muscle Activation Across the Entire Range of Motion

Analysis of the 11 studies included in this review revealed no significant difference in muscle activation levels between VRT and FWT across the entire range of motion (*p* = 0.493). The findings align with previous research conclusions (*p* = 0.082) (Andersen et al., 2020). This consistency may be due to the relationship between the training load and muscle activation (Kraemer and Ratamess, 2004). While VRT involves changing loads throughout the movement, the total load applied across the movement is similar to that of FWT. Since training load plays a crucial role in influencing muscle activation, the similarity in total load could explain the comparable activation levels.

Research comparing VRT and FWT in terms of muscle activation has shown a lack of focus on stabilizing and synergistic muscles. In this literature search, only two studies (Saeterbakken et al., 2014; Disselhorst-Klug and Williams, 2020) were found that compared the muscle activation levels of stabilizing muscles between VRT and FWT. The exercises evaluated were the squat and the bench press, targeting the erector spinae and rotator cuff muscles, with EMG results indicating no significant difference between the two training methods (*p* = 0.782). In training, attention should be given to the study of synergistic and stabilizing muscles (Disselhorst-Klug and Williams, 2020). Synergistic muscles assist the prime movers in completing movements, while stabilizing muscles are responsible for maintaining body stability and posture control (Prieske et al., 2016). These muscles are particularly important in multi-joint compound exercises. Body movements are not the result of a single muscle group but rather the coordinated work of multiple muscle groups (Palmer and McCabe, 2023). By focusing on strengthening synergistic and stabilizing muscles, the overall coordination of the body's muscles can be improved, enhancing the cooperation between different muscle groups.

### Different Proportions of Variable Resistance Load

Andersen et al. (2019) suggested that using a higher proportion of variable resistance in VRT could enhance muscle activation. This meta- analysis supports this finding, indicating that when the proportion of the variable resistance load is higher, VRT can result in greater muscle activation levels compared to FWT (*p* = 0.036). At lower proportions of variable resistance, however, the muscle activation effects of VRT did not show a significant difference from FWT (*p* = 0.433). This may be related to the training characteristics of VRT. Increasing the proportion of variable resistance in VRT leads to greater changes in the load throughout the movement (Andersen et al., 2022). Additionally, compared to FWT, the high variable resistance load may present a more novel and challenging training stimulus for athletes, potentially requiring them to recruit more muscles during the exercise (Kassiano et al., 2022).

Additionally, no significant difference was found between high and low variable resistance load proportions in VRT (*p* = 0.284). This may be due to the variation in the proportion of variable resistance loads used across different studies. In some studies, the difference in variable resistance load proportions between the two VRT methods was relatively small, which could have contributed to the lack of significant findings. Regarding the optimal proportion of variable resistance load in VRT for achieving maximum muscle activation (Andersen et al., 2019), previous research (Andersen et al., 2016) pointed out that a proportion of variable resistance comprising 35–75% of the total load is potentially optimal for effectiveness. Building on this, the current study suggests that an even higher proportion of variable resistance load in VRT could be advantageous, though the ideal proportion may vary depending on the exercise type, individual body structure, and a training level. Therefore, future research should focus on identifying the precise proportion of variable resistance load that best enhances muscle activation.

### Different Muscle Contraction Phases

This study demonstrated that VRT led to significantly higher muscle activation levels during the concentric phase compared to FWT (*p* = 0.033). These findings are consistent with previous research (van den Tillaar et al., 2022), and we additionally showed that muscle activation during the concentric phase of VRT was greater than during the eccentric phase (*p* ≤ 0.001). The comparison of muscle activation between the concentric and eccentric phases in VRT exhibited high heterogeneity.

These results may be attributed to the load characteristics of VRT. As the barbell ascends during VRT, the total load increases accordingly. Specifically, when using elastic bands as the VRT tool, the elastic coefficient increases the training load throughout the ascent, regardless of whether the bands are fixed above or below the barbell, which leads to greater joint torque at specific angles, thereby recruiting more motor units during the movement (Andersen et al., 2022). Chains, on the other hand, may provide greater instability as they lift off the ground during the ascent, further influencing muscle activation levels (Saeterbakken et al., 2016). Additionally, using elastic bands with different elastic coefficients or chains of varying weights can exacerbate the differences in muscle activation across different phases of movement in VRT, which may be a primary source of heterogeneity.

Furthermore, the differences in muscle activation across contraction phases may be related to “sticking points” that occur during the movement (van den Tillaar et al., 2024). VRT can help athletes overcome the biomechanical sticking points that often arise during FWT (Kompf and Arandjelović, 2016). One study (Saeterbakken et al., 2016) compared muscle activation levels before, during, and after the sticking point between VRT and FWT. Due to the limited research on sticking points, this meta-analysis did not focus specifically on this phenomenon. However, non-significant differences were observed in muscle activation levels between VRT and FWT before the sticking point (*p* > 0.229), whereas VRT led to higher muscle activation during and after the sticking point (*p* ≤ 0.080). In the concentric phase, where sticking points typically occur, muscles are subjected to greater loads, reaching peak activation at the end of contraction, which leads to increased muscle fatigue and subsequently higher activation levels (Kompf and Arandjelović, 2017; Kraemer and Ratamess, 2004). Since the location of sticking points varies depending on an individual's biomechanics and neuromuscular characteristics (Kompf and Arandjelović, 2016), it is challenging to pinpoint their exact position during the movement. Additionally, there is a lack of research comparing VRT and FWT in terms of muscle activation at sticking points, leaving a significant gap for future studies to determine whether VRT can consistently outperform FWT in muscle activation during and after these critical phases.

### EMG Measurement

All studies included in this research used sEMG to assess muscle activation levels. The quality of the EMG methodology in each study was evaluated using an EMG risk-of-bias assessment tool. EMG can not only monitor real-time muscle electrical signals during the movement, providing researchers with insights into muscle activation during specific movements or exercises, but also identify abnormal muscle activity patterns. This makes it a useful tool for diagnosing issues such as muscle fatigue and nerve damage, thereby aiding in the development of personalized rehabilitation programs. Additionally, EMG can be used to assess the impact of different training modalities (e.g., VRT and FWT) on muscle activation, and to evaluate whether a particular training method can enhance muscle strength and neuromuscular responses, which helps optimize training programs (Hug and Tucker, 2017).

In the included studies, detailed explanations were provided regarding signal monitoring during EMG measurement (Farago et al., 2023). However, some studies did not normalize their collected EMG data (Andersen et al., 2016, 2019; Ebben and Jensen, 2002; Saeterbakken et al., 2016). In terms of EMG signal presentation, none of the included studies reported the raw data before signal processing. Few studies reported timing-EMG data during the movement, and some did not specify whether the exercises were performed to the point of fatigue (Andersen et al., 2015, 2016; Israetel et al., 2010; Shi et al., 2024; van den Tillaar et al., 2022). These factors could potentially introduce bias in the experimental results. Moreover, there were discrepancies in the filtering methods used across different studies, and variations in exercise movements could introduce interference signals, potentially affecting study outcomes (Valentin and Zsoldos, 2016). Additionally, none of the included studies reported the participants’ body fat content, which could lead to variability in EMG measurements. Due to differences in subcutaneous fat levels, researchers should consider participants' body fat levels in EMG measurements. It is recommended that EMG data be normalized whenever possible, typically using MVC signals. Furthermore, timing-EMG should be used in studies examining different movement phases (e.g., concentric vs. eccentric contractions) to analyze muscle activation timing more precisely (Chowdhury et al., 2013).

### Application Scope

Participants in this meta-analysis were carefully selected, with all having at least six months of training experience. This makes the findings more relevant to individuals with a certain level of training background. However, those without prior training experience were not included in the analysis. Previous research (Peltonen et al., 2014) has explored muscle activation levels in untrained individuals under VRT, showing that VRT leads to higher muscle activation compared to traditional resistance training. This may be due to the increased difficulty in completing exercises with VRT. Nevertheless, only three studies (Aboodarda et al., 2011a, 2011b; Peltonen et al., 2014) reached this conclusion, focusing on exercises like bicep curls and leg presses, both of which differ from multi-joint FWT exercises in terms of muscle activation. This meta-analysis also revealed that most existing studies have primarily focused on lower-limb exercises (10 studies), with limited research comparing muscle activation between VRT and FWT in upper-body exercises (only one study).

VRT mimics isokinetic training by adjusting torque in response to changes in joint angles and lever arm length, leading to similar muscle activation patterns (Aboodarda et al., 2016). However, there are key differences between them. Isokinetic exercises require specialized equipment that provides constant velocity and precise control over movement trajectory and resistance (Christine Eyre et al., 2024). In contrast, VRT uses linearly changing resistance, generating varying torques throughout the movement (Andersen et al., 2022). Despite this, VRT is more flexible, with easily accessible and portable equipment, and can be combined with FWT to simulate sport-specific actions. Additionally, increasing the proportion of the variable resistance load throughout the movement while reducing the resistance generated in the initial phase can overcome the limitations of isokinetic training in fully activating muscles during the concentric phase (Aboodarda et al., 2016).

Furthermore, all studies included in the analysis focused on acute responses to VRT, without addressing whether long-term VRT can enhance neuromuscular system function levels. One study (Andersen et al., 2015) compared long-term VRT to long-term FWT. The results indicated that there was no significant difference between the two training methods (*p* = 0.19–0.45). However, participants in the VRT group showed a significant increase (15%) in muscle activation of the knee extensors when the knee angle was at 60° following the intervention. This suggests that VRT still holds great potential as a long-term intervention to enhance muscle activation levels. Therefore, more long-term intervention studies on the effects of VRT on muscle activation are needed, and should explore various factors such as different ages, training levels, types of athletes, training methods, joint angles, and muscle contraction phases (Stronska et al., 2022).

### Limitations and Future Directions

As the first study comparing muscle activation differences between VRT and FWT, this research presents certain limitations. The range of muscles examined was relatively narrow, with limited attention given to the activation of stabilizing and synergistic muscles. Exploring activation beyond primary muscles and investigating interactions between different muscle groups could offer more comprehensive insights for VRT application. Exercises beyond the squat, the deadlift, and the bench press also require further exploration. Variations in the proportions of variable resistance loads relative to free-weight loads across studies complicate the establishment of a standardized approach. Additionally, methods used for EMG testing, particularly the standardization of sEMG, require more rigorous attention. The participants in this study all had over six months of training experience. Whether VRT is equally effective for individuals without prior training experience remains to be investigated. Furthermore, only four studies in this meta-analysis explicitly controlled the training tempo during testing, and the lack of consistent tempo control in other studies may have introduced variability in the results due to differences in the participant’s tempo (McDonald and Bond, 2022).

## Conclusions

Throughout the entire movement process, if no specific requirements are imposed on the variable resistance load, there is no significant difference in muscle activation between VRT and FWT. However, during the concentric phase, VRT results in higher muscle activation than FWT. By increasing the proportion of the variable resistance load, VRT demonstrates superior muscle activation compared to FWT. Therefore, it is recommended to use higher proportions of the variable resistance load in daily VRT training, especially for exercises targeting the concentric phase, as VRT may lead to greater muscle activation compared to FWT.
